# Comparative evaluation of baseline characteristics of HIV mono infected and HIV/HBV co infected cohort in eastern India

**DOI:** 10.1186/1471-2334-14-S3-P14

**Published:** 2014-05-27

**Authors:** Jayeeta Sarkar, Debraj Saha, Bibhuti Saha, Bhaswati Bandyopadhyay, Runu Chakrabarty, Subhasish Kamal Guha

**Affiliations:** 1 Calcutta School of Tropical Medicine, Kolkata, West Bengal, India; 2 ICMR Virus Unit, Kolkata, ID & BG Hospital Campus, Kolkata, West Bengal, India

## Background

India has the third largest estimated number of HIV infected people and 40 million individuals in India are estimated to be Hepatitis B virus infected. In comparison to HBV mono-infection, the course of chronic HBV infection is more accelerated in HIV/HBV co-infected subjects. We analyzed the baseline characteristics of the HIV/HBV co-infected and HIV mono-infected patients.

## Methods

Between July 2011 and January 2013, a total number of 1331 HIV-seropositive treatment naïve individuals, enrolled in the ART Centre of Calcutta School of Tropical Medicine were screened for HBsAg. A number of 1253 HIV mono-infected and 78 HIV/HBV co-infected patients were characterized. The co-infected patients were evaluated for HBeAg and anti HBe antibody by ELISA. HBV DNA was detected by nested PCR amplification followed by HBV genotype determination.

## Results

The median age (35 years) was similar in HIV mono-infected and HIV/HBV co-infected group. The proportion of advanced HIV disease (WHO clinical stage III & IV) was more in HIV/HBV co-infected patients (37.1% vs. 19.9%) than HIV mono-infected subjects. The co-infected subjects had significantly higher serum bilirubin, ALT, alkaline phosphatase, creatinine and ALT/platelet ratio index. CD4 count was non significantly lower in co-infected patients. Majority (61.5%) were HBeAg reactive with higher HBV DNA (*p*=0.0001) and APRI (*p*=0.04) as compared to HBeAg negative (38.5%) patients. Genotype HBV/D (71.7%) was the predominant genotype followed by HBV/A (19.4%) and HBV/C (8.9%) among the co-infected patients from eastern India.

## Conclusion

HIV/HBV co-infection warrants long term prospective study on immune recovery, HIV and HBV viral replication, and hepatotoxicity.

